# An Own-Age Bias in Recognizing Faces with Horizontal Information

**DOI:** 10.3389/fnagi.2016.00264

**Published:** 2016-11-08

**Authors:** Andreas Schaich, Sven Obermeyer, Thorsten Kolling, Monika Knopf

**Affiliations:** Department of Psychology, Goethe UniversityFrankfurt, Germany

**Keywords:** face recognition, own-age bias, exposure duration, horizontal information, spatial frequencies

## Abstract

Horizontal information, as a result of a selective filtering process, is essential in younger adults’ (YA) ability to recognize human faces. Obermeyer et al. (2012) recently reported impaired recognition of faces with horizontal information in older adults (OA) suggesting age-variant processing. Two yet unconsidered factors (stimulus age and exposure duration) that may have influenced previous results, were investigated in this study. Forty-seven YA (18–35 years) and 49 OA (62–83 years) were tested in a 2 × 2 × 2 × 2 mixed design with the between-subjects factors age group (YA vs. OA) and stimulus age (young faces vs. older faces) and the within-subjects factors filter [filtered (HF) faces vs. unfiltered faces (UF)] and exposure duration (0.8 s vs. 8 s). Subjects were presented morph videos between pairs of faces: a starting face gradually merged into either the previously encoded target face or a control face. As expected, results showed an increase in recognition sensitivity (*d′*) with longer exposure duration in YA with both younger and older HF faces. OA, however, were unable to recognize filtered young faces not even with increased exposure duration. Furthermore, only elderly participants showed more accurate recognition with faces of their own age relative to other-age faces (own-age bias, OAB). For YA no OAB was observed. Filtered face recognition was significantly correlated with unfiltered recognition in YA but not in OA. It is concluded, that processing of horizontal information changes at a higher age. Presenting filtered or unfiltered faces both targets convergent face-specific processing only in YA but not in OA.

## Introduction

While crystallized intellectual abilities and expertise-based knowledge can be preserved until a high age (e.g., Salthouse, 1990) declining cognitive functions with age have been documented especially for working memory, attentional and executive processes (Salthouse, 1996; Craik and Salthouse, 2000; Grady and Craik, 2000). Analogous results have been gathered regarding the ability to recognize human faces (Crook and Larrabee, 1992; Searcy et al., 1999). Despite the age independent necessity to perceive, process and remember human faces on a daily basis this ability seems to develop disadvantageously over lifetime. The majority of studies depict age-dependent decline in facial recognition accuracy (Grady, 2002; Hildebrandt et al., 2013), and slower recognition processing times in OA (Grady et al., 2000). Differences in speed are assumed to rather be a product of decision making than sensory and perceptual processing speed (Pfütze et al., 2002; Habak et al., 2008). Moreover, inflated false alarm rates in OA have regularly been reported (Edmonds et al., 2012; Lee et al., 2014).

One explanation for declining face recognition performance in OA might be face specific processing mechanisms that decrease with age. Other than non-face stimuli, faces are processed primarily according to the configural information contained within them (Hole and Bourne, 2010) which can for example be demonstrated by turning a face stimulus upside-down. Yin (1969) was the first to show that face recognition is disproportionally affected by inversion: the difference in recognition accuracy with upright and inverted stimuli was much greater for faces compared with other types of objects (Face Inversion Effect, FIE). Subsequent research has shown that inversion leaves feature-based (analytic) processing relatively intact but heavily affects configural processing. This key feature in face recognition has extensively been investigated. Interestingly, OA’ ability to recognize complex stimuli like objects or scenes (analytic processing) seems to be less affected compared to recognizing faces (Park et al., 1983; Craik and Jennings, 1992; Meinhardt-Injac et al., 2014). The observed age-related decline in face recognition can, however, neither be attributed to reduced capabilities of configural face processing (Diamond and Carey, 1986; Farah et al., 1998; Meinhardt-Injac et al., 2014; Richler and Gauthier, 2014) nor to general-cognitive ability (Hildebrandt et al., 2011). Taken together, research indicates that the processing mechanisms involved in face recognition seem to be preserved with increasing age but become less efficient.

Although an aging-specific face recognition theory cannot be established to this point a number of factors have been suggested to account for differences in facial recognition between age groups. Such factors include an own-age bias (OAB) in face recognition as well as age differences in processing of horizontally aligned facial information. The OAB is characterized by preferential processing of own-age faces relative to faces of other ages (Anastasi and Rhodes, 2005; Hills and Lewis, 2011). Recent meta-analytic findings quantify differences in sensitivity due to the OAB at an effect size of *g* = 0.37 (medium effect; analogously interpretable to Cohen’s *d*) in favor of same-age compared with other age-faces (Rhodes and Anastasi, 2012). A majority of studies conducted in the past presented college-aged targets when assessing age differences and ignored the potential for superior recognition of own-age faces (Anastasi and Rhodes, 2005). The predominant account for own-age superiority in face recognition tasks has been more extensive experience or contact with a person’s own age group relative to other age groups (Rhodes and Anastasi, 2012). Corresponding empirical evidence was provided recently by Wiese et al. (2012) who reported more accurate recognition memory for older over younger faces when the OA had a high degree of daily contact with older relative to younger persons. Although only few studies are available, Rhodes and Anastasi (2012) conclude that the amount of contact measured via questionnaires appears to be related to face recognition of other ages. Ebner and Johnson (2009) for example found a positive relation (β = 0.43) between recognizing older faces and amount of contact with older adults (OA) in younger participants but no significant association recognizing younger faces and contact for OA.

A methodological approach focusing on perceptual processes in facial recognition recently proposed that the specific structure of human faces is what makes them special visual stimuli. Dakin and Watt (2009) applied a filtering process that selectively removes all visual information of an image but those restricted to certain orientation ranges and thereby simulating what information would be passed by V1 neurons (Hubel and Wiesel, 1968) tuned to a specific visual structure. The authors showed quantitative superiority in face recognition sensitivity with horizontal facial information over other alignments. Moving from horizontal to vertical, sensitivity continuously declines reaching lowest performance at vertical alignments. Moreover, those horizontal contours tend to fall into vertically aligned clusters – a phenomenon that was solely observable for faces but not for objects or natural scenes (Dakin and Watt, 2009). This clustering of horizontal visual information along a vertical axis in human faces was labeled biological ‘bar code’ and is proposed as a highly constrained one-dimensional code that makes faces special visual stimuli. Follow-up studies conducted by Goffaux and Dakin (2010) reported face specific effects for horizontal but not for vertical information as indicated by different face-specific phenomena like the FIE demonstrating that face stimuli that only contain horizontal information are processed configurally.

While Dakin and Watt (2009) measured identification accuracy of celebrity faces, Goffaux and Dakin (2010) assessed recognition performance of unfamiliar faces as indicated by target detection sensitivity (*d′*). Adding a developmental perspective, Obermeyer et al. (2012) assessed a group of younger (*M* = 21.07 years) and OAs (*M* = 66.20 years). Subjects were presented with either horizontally, vertically or unfiltered facial stimuli presented as either upright or inverted. Both age groups showed similar performance (*d′*) across five experimental conditions but considerably differed in recognizing upright faces that only contained horizontal information (YA > OA). The authors suggest that processing horizontal information may be less efficient in OA.

The present study was conducted to extend the research reported by Obermeyer et al. (2012). First, only young faces were presented to both age groups. Secondly, exposure duration to target faces was held constant at 1 s per trial. The encoding phase may have been too short for OA. The goal of this study is to assess whether OA are able to recognize horizontally filtered faces when they are provided with faces of their own age and are more familiar with the stimulus material presented. Higher accuracy with unfamiliar faces can be achieved by increasing the exposure duration to the stimulus material (Reynolds and Pezdek, 1992; Memon et al., 2003). In our study, a short encoding interval and a long encoding interval are chosen for inducing different levels of visual expertise with the stimulus material. Analogous to Obermeyer et al. (2012), face memory will be assessed presenting unfiltered faces in an encoding phase for unfamiliar faces followed by a recall phase either displaying filtered or unfiltered faces. Since faces with horizontal information are proposed to represent natural faces in a degraded form, it is being investigated whether recognizing filtered faces is associated with unfiltered face recognition for either age group. We take greater correlations between filtered and unfiltered face recognition sensitivity as evidence for underlying convergent face-specific processing.

## Materials and Methods

### Design

A mixed design was used with the between-subjects factors age group (YA vs. OA) and stimulus age (young faces vs. older faces). Filter (filtered vs. unfiltered faces) and exposure duration (0.8 s and 8 s) were both within-subject factors. Target detection sensitivity [(*d*′ = Z(hit rate) – Z(false alarm rate)] was the dependent variable (see e.g., Macmillan and Creelman, 2005).

### Subjects

A total of 47 YA (*M* = 21.89 years, *SD* = 3.27 years) participated in the study. While 23 YA (14 female) aged between 18 and 36 years (*M* = 22.16 years, *SD* = 4.26 years) were exposed to young face stimuli, 24 young subjects (14 female) aged between 19 and 26 years (*M* = 21.46 years, *SD* = 1.53 years) were presented with older faces.

Forty-nine OAs (*M* = 67.78 years, *SD* = 5.35 years) took part in the study. Twenty-seven OA (18 female) aged between 60 and 79 years (*M* = 67.22 years, *SD* = 5.32 years) were presented with young faces. Twenty-two OA (13 female) aged between 62 and 83 years (*M* = 68.45 years, *SD* = 5.44 years) were exposed to older face stimuli.

A 2 (stimulus age) × 2 (age group) between-subjects ANOVA comparing participants’ mean age confirmed an expected main effect for age group [*F*(1,92) = 2512.117, *MSE* = 192166.498, *p* < 0.001, $\eta_{p}^{2}$ = 0.965] but no differences in stimulus age [*F*(1,92) = 0.035, *MSE* = 0.701, *p* = 0.852] and no stimulus age × age group interaction [*F*(1,92) = 1.345, *MSE* = 26.856, *p* = 0.249]. Hence YA exposed to younger faces and YA exposed to older faces as well as OA presented with younger faces and OA presented with older faces were of the same age within each age group. All participants were students of Frankfurt Universities: young adults were undergraduate students of Frankfurt Goethe-University; OAs all attended the University of the Third Age, a program for education at a higher age. All participants were of Caucasian heritage and had normal or corrected-to-normal vision. Experiments were approved by the faculty ethics committee and were in line with APA guidelines according to the ethical principles of psychologists and code of conduct. Written informed consent was obtained from all participants.

### Materials

**FIGURE 1:**
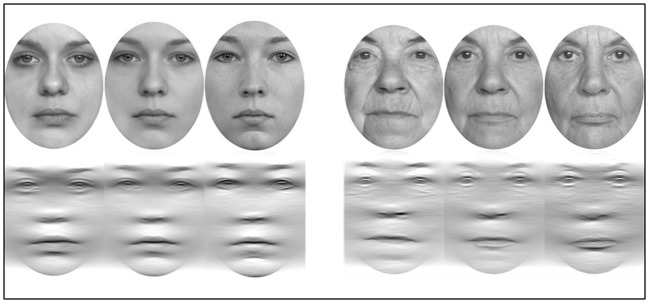
**Overview of experimental stimuli. (Upper row)** young faces (on the left) and older faces are displayed containing 0, 50, and 100% target-information. **(Lower row)** filtered young faces and filtered older faces. Face stimuli were obtained from Lindenberger et al. (2007) and Langner et al. (2010).

Two experiments were conceptualized: one version displayed young faces as stimuli, and another presented older faces (see **Figure 1**). Experiments were programmed using E-Prime 2.0 (Psychology Software Tools, Inc., Sharpsburg, PA, USA) and presented on a 22″ computer screen (LG 2210PM; resolution: 1680 × 1050) at a viewing distance of approximately 60 cm. The stimulus pool of unfamiliar faces was obtained using different databases (Minear and Park, 2004; Lindenberger et al., 2007; Langner et al., 2010). For all editing work Gimp 2.8 (The Gimp Team^1^) was used. Elliptical outer forms were cropped and converted to grayscale. The width of faces was kept constant at 400 pixels although height consistency varied slightly. Stimuli were mounted on a white 800 × 600 pixels background. Differences in contrast and luminance were equalized as best as possible and conspicuous marks, facial hair, and scars were removed.

Experiments were comprised of 64 trials (32 trials presenting unfiltered faces and 32 trials with filtered faces). In half of the trials a target was present (hits) the other half were false-alarm trials. A hit-trial was comprised of two faces: an unfamiliar face that served as a target face and a starting face that gradually merged into the target face. A false alarm trial was comprised of a third face as the starting face merged into a different face than the target face. From the entire set of faces, stimuli were assigned randomly to serve as starting faces, target faces or non-target faces. Starting faces changed on each trial. Morph continua (videos) were created using Morpheus Photo Morpher v3.16 Industrial (Morpheus Software LLC, Santa Barbara, CA, USA) with a duration of 20 s (for analogous assessment see e.g., Keenan et al., 2000; Kircher et al., 2001). The frame rate was set to 15 images per second creating a “movie-like” character. The morphing process included marking identity salient features of two faces by setting dots to similar areas (e.g., eye region: pupil, iris, lids, eye brow). The number of dots necessary for morphing two faces ranged approximately between 120 and 180 dots per morph template. As reaction times are being recorded by the computational software during experimental procedure, for data analysis, individual mean morph levels for particular conditions were converted from milliseconds to percentages with greater numbers indicating more target information along the morph continuum. Stimuli displaying only horizontally aligned information were generated using Matlab 7.13 (The Mathworks, Inc., Natick, MA, USA). The filtering process includes breaking down a stimulus to its basic components by Fourier transforming it and multiplying the Fourier energy with an orientation filter (wrapped Gaussian profile with a standard deviation of 20°) allowing only horizontal information to pass (for further details see Dakin and Watt, 2009).

To screen for cognitive function, subjects completed the WAIS-R Digit-Span subtest (Tewes, 1991/1994). The WAIS-R Digit-Span subtest involves remembering growing chains of numbers forward and backward and assesses working memory. Participants answered questions related to degree of social contact with younger (18–30 years) and OAs (60–80 years) analogous to Wiese et al. (2012). Subjects were asked to indicate the amount of time they spend with each age group (hours per week) as well as the number of different contact persons. The questions were preceded by a short explanation asking subjects to only consider people they are familiar with.

### Procedure

**FIGURE 2:**
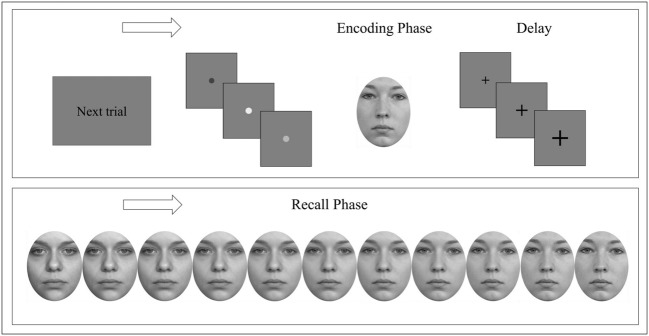
**Experimental procedure.** A single trial consisted of a go-signal followed by an encoding phase of a target face presented for either 0.8 s or 8 s. Subsequent delay was accompanied by a fixation cross that became enlarged twice. Each recall phase started with a different face either merging into the previously presented target face or a (different) control face. Participants were instructed to respond as soon as they recognized the previously presented target face but to show no reaction in case of target absence. Face stimuli were obtained from Langner et al. (2010).

The experiment took approximately 30 min and could be aborted by the subject at any time. All participants completed the experiment. Trials were presented randomly – **Figure 2** displays an experimental target trial. Subjects were instructed to press the space bar with their dominant hand of a standard keyboard as soon as they recognized a target but to show no reaction in case of target absence for each session. No feedback was given. Both, accuracy and recognition with less target information were stressed without emphasizing either.

Each trial was followed by an intertrial interval of 3.5 s before presenting a new learning face followed by a fixation cross that became enlarged twice (6 s total). To familiarize subjects with the task participants trained with a set of stimuli that were not presented during the test session. After completing the experiment the individual digit-span was assessed.

## Results

### Preliminary Analysis

Prior to the main analysis, in a first step, differences in general cognitive function and social contact with younger and older persons were being investigated. Since our task required participants to respond to a starting face that gradually merged into either the previously encoded target face or a control face there may have been differences considering the amount of target information necessary for making a familiar judgment between the different groups. Whether YA and OA required equal amounts of target face information was being analyzed in a second step. A 2 (age group) × 2 (stimulus age) between-subjects ANOVA was conducted analyzing the digit-span results. Three subjects (2 YA) did not complete the digit-span assessment and contact questionnaire due to a shortage of time. Those subjects were, however, included in the main analysis. There were no differences for age group [*F*(1,89) = 1.276, *MSE* = 9.164, *p* = 0.262], or stimulus age [*F*(1,89) = 1.607, *MSE* = 11.547, *p* = 0.208]. An age group × stimulus age interaction was not obtained [*F*(1,89) = 0.151, *MSE* = 1.812, *p* = 0.617].

Social contact was measured as the time spent with other persons and as the number of contact persons. Both groups reported more contact with their own age group in terms of time and number of persons. A 2 (age group) × 2 (stimulus age) × 2 (contact age: time spent with YA vs. time spent with OA) mixed-design ANOVA analyzing the time spent with younger and older persons yielded main effects for age group [YA > OA; *F*(1,89) = 55.533, *MSE* = 16752.329, *p* < 0.001, $\eta_{p}^{2}$ = 0.384] and contact age indicating more contact with younger persons [*F*(1,89) = 40.392, *MSE* = 11978.961, *p* < 0.001, $\eta_{p}^{2}$ = 0.312] but not for stimulus age [*F*(1,89) = 1.600, *MSE* = 482.713, *p* = 0.209]. Decomposition (*p*s Bonferroni corrected for multiple comparisons; *p*_crit_ = 0.0083) of a significant contact age x age group interaction [*F*(1,89) = 147.522, *MSE* = 43749.574, *p* < 0.001, $\eta_{p}^{2}$ = 0.624] yielded more contact with participants’ own age compared to the other age [*M_YA contact YA_* = 55.82, *SD* = 23.44, *M_OA contact YA_* = 4.97, *SD* = 5.62, *t*(92) = 13.882, *p* < 0.001, *d* = 3.36; *M_YA contact OA_* = 7.96, *SD* = 10.08, *M_OA contact OA_* = 19.00, *SD* = 22.35, *t*(92) = 3.061, *p* = 0.003, *d* = 0.38], within either age group greater contact with the own age [*M_YA contact YA_* = 55.82*, SD* = 23.44, *M_YA contact OA_* = 7.96, *SD* = 10.08, *t*(45) = 12.151, *p* < 0.001, *d* = 2.74; *M_OA contact YA_* = 4.97, *SD* = 5.62, *M_OA contact OA_* = 19.00, *SD* = 22.35, *t*(46) = 4.226, *p* < 0.001, *d* = 1.00], and greater age-congruent contact for YA over OA [*M_YA contact YA_* = 55.82*, SD* = 23.44, *M_OA contact OA_* = 19.00, *SD* = 22.35, *t*(92) = 7.429, *p* < 0.001, *d* = 1.52] but no differences for age-incongruent contact between YA and OA [*M_YA contact OA_* = 7.96, *SD* = 10.08, *M_OA contact YA_* = 4.97, *SD* = 5.62, *t*(92) = 1.771, *p* = 0.080].

Regarding the number of persons subjects have contact with per week significant main effects for contact age [number of younger persons > number of older persons; *F*(1,89) = 12.561, *MSE* = 2270.678, *p* < 0.001, $\eta_{p}^{2}$ = 0.124] and age group [OA > YA; *F*(1,89) = 6.091, *MSE* = 1636.557, *p* = 0.015, $\eta_{p}^{2}$ = 0.064] were found but not for stimulus age [*F*(1,89) = 0.069, *MSE* = 18.446, *p* = 0.794]. There was a likewise significant age group x contact age interaction [*F*(1,89) = 48.386, *MSE* = 8684.409, *p* < 0.001, $\eta_{p}^{2}$ = 0.352]. Decomposition of this interaction yielded the same pattern as before: more contact with participants’ own age compared to the other age [*M_YA contact YA_* = 25.17, *SD* = 27.02, *M_OA contact YA_* = 5.52, *SD* = 5.41, *t*(92) = 4.888, *p* < 0.001, *d* = 1.21; *M_YA contact OA_* = 4.46, *SD* = 4.98, *M_OA contact OA_* = 12.32, *SD* = 10.02, *t*(92) = 4.776, *p* < 0.001, *d* = 1.05], within either age group greater contact with subjects’ own age [*M_YA contact YA_* = 25.17*, SD* = 27.02, *M_YA contact OA_* = 4.46*, SD* = 4.98, *t*(45) = 5.768, *p* < 0.001, *d* = 1.30; *M_OA contact YA_* = 5.52, *SD* = 5.41, *M_OA contact OA_* = 12.32, *SD* = 10.02, *t*(6) = 4.348, *p* < 0.001, *d* = 0.88], and greater age-congruent contact for YA compared to OA [*M_YA contact YA_* = 25.17, *SD* = 27.02, *M_OA contact OA_* = 12.32, *SD* = 10.02, *t*(92) = 3.108, *p* = 0.002, *d* = 0.69] but no differences for age-incongruent contact between YA and OA [*M_YA contact OA_* = 4.46*, SD* = 4.98, *M_OA contact YA_* = 5.52, *SD* = 5.41, *t*(92) = 0.880, *p* = 0.381].

Next the amount of target information necessary for making a familiar judgment was analyzed. As 4 OA and 1 YA were not able to respond correctly to any target trial in specific filtered conditions, no amount of target information was being recorded for these individuals and they were therefore not included in the target information analysis. A mixed-model ANOVA [2 (age group) × 2 (stimulus age) × 2 (filter) × 2 (exposure duration)] with age group and stimulus age as between measures and filter as well as exposure duration as within-subjects factors was conducted to analyze the results for target information. No main effects were obtained [age group *F*(1,87) = 3.429, *MSE* = 1024.142, *p* = 0.067, stimulus age *F*(1,87) = 2.238, *MSE* = 668.586, *p* = 0.138, filter *F*(1,87) = 3.131, *MSE* = 319.959, *p* = 0.080, exposure duration *F*(1,87) = 1.260, 92.651, *p* = 0.265] and no interactions indicating that YA and OA in either experimental condition (younger vs. older faces) did not differ regarding the amount information necessary for making a familiar judgment.

### Main Analysis

In a first step a mixed-model ANOVA [2 (age group) × 2 (stimulus age) × 2 (filter) × 2 (exposure duration) with age group and stimulus age as between-subjects factors and filter as well as exposure duration as within-subjects factors was conducted analyzing the results for sensitivity (**Table 1**). Significant interactions were decomposed running multiple Bonferroni corrected comparisons.

**Table 1:** Summary of means and standard deviations for sensitivity, false alarms, target information, and digit span.

	Young faces		Older faces	
	YA	OA	YA	OA
		
	*M (SD)*	*M (SD)*	*M (SD)*	*M (SD)*
**Sensitivity (*d’*)**				
HF 0.8 s	0.62 (1.16)	0.25 (0.66)	0.88 (0.99)	0.42 (0.70)
HF 8 s	0.92 (0.81)	0.23 (0.56)	1.29 (1.06)	1.17 (0.63)
UF 0.8 s	1.74 (1.16)	1.12 (1.00)	1.70 (0.88)	0.97 (0.87)
UF 8 s	2.73 (1.35)	1.94 (1.17)	2.96 (1.33)	2.23 (0.91)
**False alarms**				
HF 0.8 s	0.32 (0.24)	0.36 (0.23)	0.39 (0.25)	0.40 (0.21)
HF 8 s	0.31 (0.31)	0.34 (0.34)	0.41 (0.41)	0.33 (0.33)
UF 0.8 s	0.17 (0.17)	0.24 (0.24)	0.35 (0.35)	0.28 (0.28)
UF 8 s	0.14 (0.14)	0.16 (0.16)	0.28 (0.28)	0.23 (0.23)
**Target information**				
HF 0.8 s	66.96 (16.05)	66.53 (12.38)	70.59 (15.24)	67.77 (10.11)
HF 8 s	70.98 (16.02)	66.54 (11.75)	74.53 (12.42)	69.60 (10.84)
UF 0.8 s	71.85 (8.17)	67.93 (11.82)	72.22 (12.30)	73.11 (8.17)
UF 8 s	71.53 (7.11)	65.25 (9.13)	73.19 (10.47)	73.42 (7.27)
**Digit span**	11.70 (2.20)	11.35 (2.58)	12.68 (2.87)	11.77 (3.04)

*YA, young adults; OA, older adults; HF, horizontally filtered faces; UF, unfiltered faces.*

Given our *a priori* predictions, the obtained data were then analyzed separately for each age group in a second step. The ANOVA indicated significant main effects for age group [YA > OA: *F*(1,92) = 18.838, *MSE* = 30.591, *p* < 0.001, $\eta_{p}^{2}$ = 0.170], stimulus age [older faces > younger faces: *F*(1,92) = 4.000, 6.496, *p* = 0.048, $\eta_{p}^{2}$ = 0.042], as expected greater recognition for unfiltered stimuli compared to filtered faces [*F*(1,92) = 249.312, *MSE* = 137.662, *p* < 0.001, $\eta_{p}^{2}$ = 0.730], and a significant main effect for exposure duration [8 s > 0.8 s: *F*(1,92) = 48.972, *MSE* = 49.623, *p* < 0.001, $\eta_{p}^{2}$ = 0.347].

Three significant two-way interactions were obtained (interactions involving more than two factors did not reach significance). First, a filter × stimulus age interaction [*F*(1,92) = 5.286, *MSE* = 2.919, *p* = 0.024, $\eta_{p}^{2}$ = 0.054] indicates that differences between recognizing filtered and unfiltered faces is greater in younger than in older face stimuli. Second, a significant filter x exposure duration interaction was obtained [*F*(1,92) = 18.972, *MSE* = 12.404, *p* < 0.001, $\eta_{p}^{2}$ = 0.171] which indicates that an increase in exposure duration has a greater impact on sensitivity to unfiltered faces than on recognizing filtered stimuli. Decomposition of both interactions is illustrated in **Table 2**.

**Table 2:** Summary of decomposed interactions from sensitivity analysis.

	*M* (*SD*)	*M* (*SD*)	*df*	*t*	*p*^(a)^	*d*
**Filter × Stimulus Age**						
HF YF – UF YF	0.48 (0.65)	1.85 (0.93)	49	12.892	<0.001	1.73
HF YF – HF OF	0.48 (0.65)	0.94 (0.63)	95	3.529	0.001	0.72
UF YF – UF OF	1.85 (0.93)	1.98 (0.88)	95	0.700	0.486	0.14
HF OF – UF OF	0.94 (0.63)	1.98 (0.88)	45	9.279	<0.001	1.36
**Filter × Exposure**						
HF 0.8s – HF 8.0 s	0.54 (0.91)	0.88 (0.88)	95	2.848	0.005	0.38
UF 0.8s – UF 8.0 s	1.38 (1.03)	2.45 (1.25)	95	7.473	<0.001	0.94
HF 0.8s – UF 8.0 s	0.54 (0.91)	1.38 (1.03)	95	8.208	<0.001	0.87
HF 8.0s – UF 8.0 s	0.88 (0.88)	2.45 (1.25)	95	12.847	<0.001	1.47

*^(a)^Corrected type I error (α = 0.05/4 = 0.025); HF, horizontally filtered faces; UF, unfiltered faces; YF, young faces; OF, older faces; d, Cohen’s d.*

**FIGURE 3:**
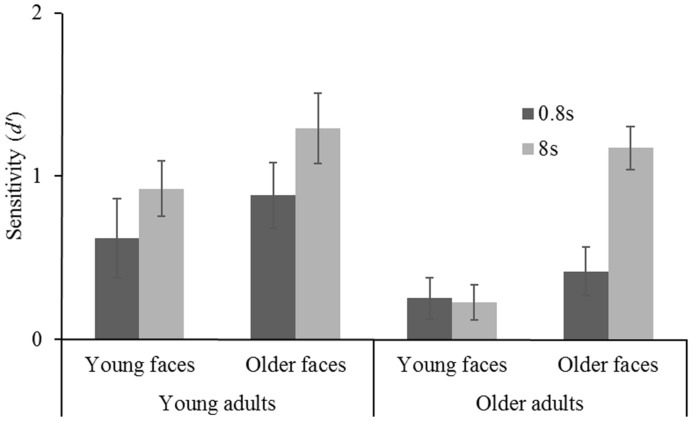
**Face recognition performance (*d*′) for short (0.8 s) and long (8 s) exposure duration as a function of stimulus age (younger faces vs. older faces) for young adults and older adults.** Error bars represent standard errors.

Finally, a significant filter × age group interaction [*F*(1,92) = 4.119, *MSE* = 2.274, *p* = 0.045, $\eta_{p}^{2}$ = 0.043] indicated, as expected, differences in sensitivity to filtered and unfiltered conditions between both age groups. This interaction will be further analyzed in the following. First, a mixed-design ANOVA [2 (age group) × 2 (stimulus age) × 2 (exposure duration)] was conducted testing for differences in sensitivity to unfiltered faces. There were significant main effects for age group [YA > OA: *F*(1,92) = 17.330, *MSE* = 24.774, *p* < 0.001, $\eta_{p}^{2}$ = 0.159] and exposure duration [8 s > 0.8 s: *F*(1,92) = 55.930, *MSE* = 55.824, *p* < 0.001, $\eta_{p}^{2}$ = 0.378], but no difference whether younger or older faces were presented [*F*(1,92) = 0.247, *MSE* = 0.353, *p* = 0.620]. Both age groups profited considerably from longer exposure duration, however, there were no biases toward own-age faces (as indicated by non-existent interactions). An analogous analysis testing for differences in sensitivity to filtered faces (**Figure 3**) indicated similar results for age group [YA > OA: *F*(1,92) = 10.839, *MSE* = 99.822, *p* = 0.001, $\eta_{p}^{2}$ = 0.105] and exposure duration [8 s > 0.8 s: *F*(1,92) = 9.273, *MSE* = 6.204, *p* = 0.003, $\eta_{p}^{2}$ = 0.092]. Additionally, a significant main effect for stimulus age was obtained [*F*(1,92) = 12.138, *MSE* = 9.061, *p* < 0.001, $\eta_{p}^{2}$ = 0.117]. A triple interaction between age group, stimulus age, and exposure duration was not significant [*F*(1,92) = 2.026, *MSE* = 1.355, *p* = 0.158, $\eta_{p}^{2}$ = 0.022].

### Testing for an Own-Age Bias

Considering that our *a priori* hypotheses concerned the question whether OA are able to recognize filtered faces when exposure duration is increased and old stimuli are added rather than solely presenting young faces, separate analyses for either age group were conducted testing for an own-age-bias with filtered faces. For YA there were no main effects [exposure duration *F*(1,45) = 3.313, *MSE* = 3.003, *p* = 0.075, stimulus age *F*(1,45) = 2.070, *MSE* = 2.362, *p* = 0.157] and no interaction [*F*(1,45) = 0.073, *MSE* = 0.066, *p* = 0.788]. For OA, however, both main effects were significant [exposure duration *F*(1,47) = 7.256, *MSE* = 3.204, *p* = 0.010, $\eta_{p}^{2}$ = 0.134, stimulus age *F*(1,47) = 20.194, *MSE* = 7.452. *p* < 0.001, $\eta_{p}^{2}$ = 0.301] and a significant exposure duration x stimulus age interaction was obtained [*F*(1,47) = 8.360, *MSE* = 3.691, *p* = 0.006, $\eta_{p}^{2}$ = 0.151].

This interaction was decomposed further running multiple comparisons (*p*_crit_ = 0.0125, for descriptive statistics; **Table 1**). There was no difference between sensitivity to young faces and older faces when exposure duration was short [*t*(47) = 0.843, *p* = 0.403]. A large effect, however, was obtained comparing sensitivity to young and older faces with long exposure duration [*t*(47) = 5.553, *p* < 0.001, Cohen’s *d* = 1.59]. This shows that an OAB toward recognizing filtered faces is observable in OA when exposure duration is long. Furthermore, the impact of a higher exposure duration interval was considerable on recognizing older faces [*t*(21) = 3.245, *p* = 0.004, Cohen’s *d* = 1.13] but did not show in sensitivity to young faces [*t*(26) = 0.174, *p* = 0.864].

Since inflated false alarm rates have previously been reported in OA such an analysis was conducted. A mixed-model ANOVA [2 (age group) × 2 (stimulus age) × 2 (filter) × 2 (exposure duration)] indicated that OA made more false alarms than YA [*F*(1,92) = 6.526, *MSE* = 0.612, *p* = 0.012, $\eta_{p}^{2}$ = 0.066], as well as significant main effects for filter [filtered faces > unfiltered faces: *F*(1,92) = 36.513, *MSE* = 1.557, *p* < 0.001, $\eta_{p}^{2}$ = 0.284], and exposure duration [shorter duration > longer duration: *F*(1,92) = 5.737, *MSE* = 0.160, *p* < 0.019, $\eta_{p}^{2}$ = 0.059] but not for stimulus age [*F*(1,92) = 0.006, *MSE* = 0.001, *p* = 0.936]. Since no interactions were obtained, differences in false alarms were not pursued any further.

**FIGURE 4:**
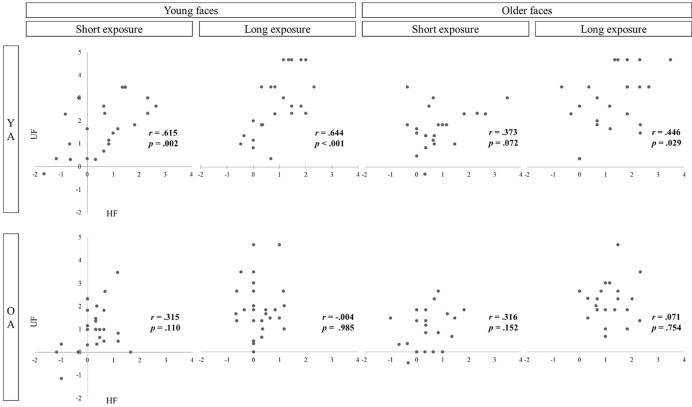
**Associations between sensitivity to filtered (HF) and unfiltered (UF) face recognition as a function of age group (YA vs. OA), stimulus age (younger vs. older faces), and exposure duration (short vs. long exposure)**.

Lastly, correlations coefficients (Pearson product-moment correlation, data are plotted in **Figure 4**) between filtered and unfiltered face recognition were calculated. There were three significant associations as well as one marginal correlation observable in YA [young faces: *r*_8s_(21) = 0.615, *p* = 0.002, *r*_8s_(21) = 0.644, *p* < 0.001; older faces: *r_0._*_8s_(22) = 0.373, *p* = 0.072, *r*_8s_(22) = 0.446, *p* = 0.029] but none in OA [young faces: *r_0._*_8s_(25) = 0.315, *p* = 0.110, *r*_8s_(25) = -0.004, *p* = 0.985; older faces: *r_0._*_8s_ (20) = 0.316, *p* = 0.152, *r*_8s_(20) = 0.071, *p* = 0.754].

## Discussion

The present study was conducted to extend previous findings regarding young and OAs’ ability to process faces that only contain horizontal information by two factors. First, stimulus age was introduced: YA and OA were presented with either younger or older stimuli resulting in a fully crossed design allowing assessment of an OAB which is expressed in greater sensitivity to age-congruent stimuli as opposed to age-incongruent faces. Second, a variation in exposure duration was introduced by providing subjects with both short and long encoding intervals. We found an own-age-bias in OAs’ sensitivity to faces with horizontal information. Specifically, the OAB was only observable when exposure duration was long. YA’ face recognition performance, however, was not affected by presenting younger or older filtered stimuli. This age-variant result was furthermore only observable when filtered faces had to be recognized. When recognizing unfiltered faces exposure duration and stimulus age had equal effects on both age groups. This finding adds to the notion that YA and OA do indeed process horizontal information differently (Obermeyer et al., 2012).

While the obtained main effects for filter and exposure duration were anticipated, the main effect for stimulus age as well as greater sensitivity to filtered older stimuli compared to filtered young stimuli were unexpected. It is hypothesized that this result is a product of the selective filtering process which may have a positive impact on older facial stimuli compared to younger faces. First, it seems plausible to assume that older faces in general contain more information compared to younger faces. Specifically, older faces differ from younger faces concerning cues of aging like wrinkles and skin tightness. Those features are likely to have passed the selective filtering process (Please compare filtered young and older faces of **Figure 1**) and may have added identity salient cues only to the older face stimuli. Consequently, recognition of filtered older faces may have been easier compared to younger stimuli. This factor may, however, have had a different impact on either age group as we will discuss below.

To further test for differences concerning the role of horizontal information in both age groups, it was investigated whether sensitivity to filtered faces is associated with unfiltered face recognition. We took high correlations as indicators for targeting similar face specific processes. Results showed that processing of horizontal information was especially efficient in YA when presented with age-congruent face stimuli. The impact of processing filtered faces on recognizing unfiltered faces was smaller when presented with older faces. An increase in exposure duration, however, was accompanied by a greater association similar to recognizing younger faces. When exposure duration was longer processing of horizontal facial cues was correlated with unfamiliar face recognition in younger adults (YA) regardless of stimulus age. However, the impact of filtered face processing on unfiltered recognition was characterized by a completely different pattern in OA. With longer exposure duration, OA’ processing of horizontal information became more inefficient. Decomposing the factor stimulus age did not provide additional information about OA’s ability to process horizontal information as the pattern of correlations was similar for recognizing younger or older faces. Especially when exposure duration was long, sensitivity to horizontal faces had no impact on unfiltered face recognition.

There are several potential explanations for the magnitude (Cohen’s *d* = 1.59) of the obtained OAB in OA with filtered faces (and the absence of an OAB in YA). Among those, previously argued cues of aging as well as the absence of correlations between filtered and unfiltered recognition in OA have to be considered. First, visual aging cues might be perceived and or processed differently by YA and OA in general. Individuals belonging to certain groups and therefore sharing a common face space (e.g., same age or same ethnic background) are likely to be more sensitive to detecting certain facial features that are specific to that group. One example to this thought is a study by Hu et al. (2014) who recently showed that both children and adults scan faces of own and other races differently. Both age groups fixated the eyes of Caucasian faces significantly longer than the eyes of Chinese faces. Conversely, the Chinese participants scanned the mouth and nose region of Chinese faces more extensively than the corresponding areas of Caucasian faces. Following that line of thought we hypothesize that OA may especially attend to aging cues when recognizing faces and/or be therefore more efficient in processing this source of diagnostic information. Certainly, YA may be susceptible to the proposed aging cued feature-based processing as a result of the selective filtering process in a similar manner as OA are. It, however, seems plausible that this proposed effect has a greater impact on OA compared to YA. Future research should therefore assess the impact on both YA and OA and to what degree it might account for the OAB. Another approach for future studies would be to compare sensitivity to older stimuli with aging cues eliminated that are not part of the general Gestalt of older faces (configural processing) with sensitivity to the same stimulus set containing all information (including natural aging features). Additionally, horizontal filter could be added as a factor which would allow quantifying the impact of aging cues in filtered vs. unfiltered older faces. A somewhat similar approach has already been pursued recently. Examining aftereffects with hybrid images that combined the structure and shape of younger, older, and same age celebrity faces Lai et al. (2013) showed that shape and texture contribute differently to different face representations, with texture dominating for age and that encoding of shape and texture seem to occur separately. As only YAs were assessed in this study future research should focus on assessing OAs with an analogous procedure.

Secondly, the obtained OAB in OA but not in YA with filtered faces might be the manifestation of different face processing mechanisms used by either age group. Since configural processing is the key feature in (unfiltered) face recognition, it is plausible to assume that the obtained associations between filtered and unfiltered recognition in YA primarily reflects this ability. As OAs likewise rely on configural processing when recognizing faces (e.g., Meinhardt-Injac et al., 2014) the absence of correlations between filtered and unfiltered conditions in OA in our study may be due to different mechanisms being targeted with filtered and unfiltered faces. In other words, it is speculated that OA do not perceive a holistic face (to the same degree as YA do) when presented with faces that only contain horizontal information.

A third finding that adds to understanding age differences in recognizing filtered faces are the obtained results of OA concerning younger faces. As shown in **Figure 3**, an increase in exposure duration did not have any impact on OA’ sensitivity to younger filtered faces remaining slightly above chance level. Most likely, OA were simply not able to extract identity-diagnostic information from filtered younger faces. The question arises, why OA were able to recognize older filtered faces at the same performance level as YAs with perception of the whole face disrupted when exposure duration was long? We suggest that OA’ increase in performance with increased exposure duration with filtered older faces indicates a switch to analytic processing. Additionally, as discussed above, this type of part-based processing might be particularly efficient in OA when it comes to processing older faces. An increase of analytic processing with increased exposure duration has previously been reported. Although only very little research systematically manipulated exposure duration of the study faces, Hole (1994) showed that with longer exposure duration participants switched to a feature-matching strategy as opposed to configural processing under short presentations.

Our hypothesis that OA do not actually perceive faces when confronted with filtered stimuli is moreover supported by the repeated observation of older participants reporting that they were unable to recognize anything, when initially confronted with filtered faces prior to the experiment. Two recent publications add to understanding the role of horizontal information in face recognition. Balas et al. (2015) tested 5–10 year olds with faces or objects (houses) that were either presented upright or inverted. Stimuli either contained vertical, horizontal or both vertical and horizontal information. Results showed slower reaction times to vertically filtered images than horizontally filtered images in faces but not in houses. Furthermore, older children were more likely to show such biased face detection for horizontal information than younger children. At the ages of 5–8, however, there seems to be no such bias in response time to faces that contained horizontal information suggesting development in middle childhood. Goffaux et al. (2015) recently reported convergent results testing subjects aged from 6 to 74 years of age applying a method similar to Balas et al. (2015) presenting subjects likewise with faces that either contained vertical, horizontal or both vertical and horizontal information. Face specific processing was inferred based on the FIE which was significant with faces that contained horizontal information for the age groups 12–13, 14–15, 16–17, 18–19, 20–35, and >59 years of age. At the ages of 6–7, 8–9, and 10–11 years no FIE for horizontal information was observed which in line with the results presented by Balas et al. (2015) suggesting progressive maturation of horizontal processing until young adulthood. At elderly adulthood, however, FIE development with faces that contained only horizontal information dropped notably with a non-linear function best fitting the FIE as a function of age. This finding is in line with Obermeyer et al. (2012) suggesting that OAs process horizontal information differently than YAs. Our results reported here add to this notion while offering a new scope to the role of horizontal information in YA and OA’ face processing as for the first time stimulus age was being investigated systematically.

Whether the obtained correlation coefficients in YA but not in OA represent convergent configural face recognition ability cannot exhaustively be concluded from our data. Therefore, future research needs to assess whether OA’ ability to recognize older faces with horizontal information is actually based on face processing mechanisms (i.e., face-specific configural processing) or whether low-level selective feature-based processing is primarily being targeted. This could be accomplished by applying an experimental approach similar to Goffaux and Dakin (2010) testing for the behavioral (face specific) signature of horizontally filtered and unfiltered age congruent-faces in OAs. Additionally, we propose that future studies add faces that are familiar to the subjects like it was initially done by Dakin and Watt (2009). Subsequent research primarily focused on perceptual processes assessing unfamiliar face recognition. To this point it remains unclear, whether OA are able to identify filtered faces they are familiar with which would involve accessing long term memory representations.

## Author Contributions

AS: conception and design of the work, data collection, data analysis and interpretation, drafting the article, critical revision of the article, final approval of the version to be published. SO: conception and design of the work, critical revision of the article, final approval of the version to be published. TK: conception and design of the work, critical revision of the article, final approval of the version to be published. MK: conception and design of the work, critical revision of the article, final approval of the version to be published.

## Conflict of Interest Statement

The authors declare that the research was conducted in the absence of any commercial or financial relationships that could be construed as a potential conflict of interest.

## Footnotes

1 www.gimp.org
